# Acupuncture for Low Back Pain: Reevaluation of Systematic Reviews and Meta-analyses

**DOI:** 10.1007/s11916-023-01139-w

**Published:** 2023-07-11

**Authors:** Wen-xi Yan, Hui-ze Lin, Xiang Wang, Wei Zhang, Lan-ping Liu, Jin-na Yu, Tao Yang

**Affiliations:** Department of Acupuncture-Moxibustion, Guang’anmen Hospital, China Academy of Chinese Medical Sciences, Beijing, 100053 China

**Keywords:** Acupuncture, Reevaluation, AMSTAR 2, PRISMA, GRADE

## Abstract

**Purpose of Review:**

This overview aims to reevaluate the methodological quality, report quality, and evidence quality of systematic reviews (SRs)/meta-analyses (MAs) of acupuncture for low back pain to determine whether acupuncture effectively treats low back pain (LBP).

**Recent Findings:**

Twenty-three SRs/MAs were deemed eligible for the present overview. The AMSTAR 2 score showed that the methodological quality of one SR/MA was of medium quality, one was of low quality, and 21 were of critically low quality. Based on the results of the PRISMA evaluation, there are certain areas for improvement in the quality of reporting of SRs/MAs. There were some reporting flaws in the topic of search strategy (8/23, 34.78%), certainty assessment (4/23, 17.39%), the certainty of evidence (4/23, 17.39%), registration and protocol (3/23, 13.04%), and availability of data, code, and other material (1/23, 4.35%). Results from the GRADE evaluation indicated that 13 of 255 outcomes were rated as moderate, 88 were low, and 154 were very low.

**Summary:**

Acupuncture effectively treated LBP in the SRs/MAs included in the reevaluation. However, the methodological, report, and evidence-based quality of the SRs/MAs on acupuncture for LBP was low. Therefore, further rigorous and comprehensive studies are warranted to improve the quality of SRs/MAs in this field.

## Introduction

Low back pain (LBP) typically manifests as pain, stiffness, or muscle tension below the costal margin and above the inferior gluteal fold with or without sciatica (pain radiating down the leg from the lower back) [1, 2]. Rarely is a specific cause of low back pain identifiable. Therefore, most LBP is considered non-specific [3]. Non-specific low back pain (NLBP) has become a significant problem in health care. It is estimated that 84% of people will experience low back pain at some point before they die, roughly 23% will suffer chronic low back pain, and 11 to 12% will be disabled as a result of the pain [4]. Low back pain is classified according to its duration. Chronic LBP lasts more than 12 weeks, subacute LBP with a course of 2~4 weeks, and acute LBP with less than 4 weeks [5]. Acupuncture has been recommended in several guidelines for acute and chronic low back pain [6–8]. Despite studies suggesting the positive effects of acupuncture on low back pain, original trials are of low quality. There is insufficient evidence to draw definitive conclusions about acupuncture’s effectiveness.

Several systematic reviews (SRs) and meta-analyses (MAs) of acupuncture for low back pain have been conducted [9]. To overcome the limitations of an individual SR/MA and provide extensive evidence, an overview of SRs/MAs is required [10]. According to our knowledge, an overview of acupuncture’s efficacy and safety in treating low back pain has yet to be published. Therefore, in order to assess the most recent data and draw conclusions, we included studies published from the establishment of the databases to December 2022 and evaluated the methodological quality and outcome measures of SRs/MAs on acupuncture for low back pain using AMSTAR 2 (A Measurement Tool to Assess Systematic Reviews, revised edition), to assess the quality of reports from the included SRs/MAs by PRISMA statement (Preferred Reporting Items for Systematic Reviews and Meta-Analyses), to grade the quality of evidence by GRADE system (The Grading of Recommendations Assessment, Development and Evaluation), and to summarize the conclusions of these SRs and MAs to further clarify the effectiveness and safety of acupuncture for low back pain [11]. 

## Methods

### Inclusion Criteria

#### Study Design

SRs/MAs based on randomized control trials (RCTs) in which the participants with LBP were diagnosed according to internationally recognized clinical guidelines without restrictions on gender, age, race, duration, intensity, condition, or source.

#### Study Intervention

Experimental group intervention included various acupuncture therapies (acupuncture, electroacupuncture, auricular acupuncture, warm acupuncture, fire acupuncture, etc.) and acupuncture combined with other therapies.

#### Study Comparison

Control group intervention could be western medicine, physical therapy, manipulation, massage, conventional therapy, traditional Chinese medicine, placebo acupuncture, sham acupuncture, usual care, transcutaneous electrical nerve stimulation (TENS), waiting list, or different acupuncture treatment.

#### Types of Outcome Measures

##### Primary Outcomes

1. Pain intensity (measured with a Visual Analogue Scale (VAS), Japanese Orthopaedic Association Scores (JOA), Numerical Rating Scale (NRS or NPRS), etc.)
2. The dysfunction scale: Roland-Morris Disability Questionnaire (RMDQ); Oswestry Disability Index (ODI), Pain Disability Index (PDI), Hanover Functional Ability Questionnaire (HFAQ);
3. Assessment of therapy effectiveness: total effective rate/effective ratio, recurrence rate, excellent rate, total clinical efficacy, recovery rates;

##### Secondary Outcomes

1. Pain-related outcomes (measured with a Brief Pain Inventory(BPI), Von Korff Chronic Pain Grade Scale (CPGS), McGill Pain Questionnaire (MPQ), Pain Rating Scale (PRS), Brief Pain Inventory Short Form (BPI-SF), Numbers of Pills (NOP), etc.)
2. Dysfunction examination: Schober test, range of motion (ROM), lumbar flexion range (LFR);
3. The quality of life: the short-form-12 health survey (SF-12), The short-form-36 health survey (SF-36), EuroQol 5D (EQ-5D).

### Exclusion Criteria

Non-RCT SRs/MAs, review comments, conference abstracts, editorials, guidelines, and studies on which the data could not be extracted.

Intervention of experimental group was not acupuncture as above.

Literature not published in Chinese or English.

Duplicate published literature.

### Search Strategy

This study searched the following databases: PubMed, Embase, Cochrane Library, Web of Science, Chinese Biomedical Literature Database (CBM), the China National Knowledge Infrastructure (CNKI), the VIP Database, and the WanFang Database. All online databases were searched from the establishment of the databases to December 2022. The following grouped terminology was used for searching: (“Low back pain” OR “Non-specific low back pain” OR “Non-specific low back pain” OR “Lower Back Pain” OR “LBP” OR “NLBP” OR “Acute lumbar sprain”) AND (“Acupuncture” OR “Electroacupuncture” OR “Manual acupuncture” OR “Filiform needle” OR “Acupuncture point” OR “Acupoint” OR “Auricular acupuncture” OR “Warm acupuncture” OR “Fire acupuncture”) AND (“Meta-analysis” OR “Systematic review”). Furthermore, the bibliographies of these papers, conference papers, and published journal bibliographies were also retrieved to ensure that pertinent information would not be missed. According to the researchers’ languages, retrieval was limited to English and Chinese. Furthermore, experts in the field were consulted.

The search strategy in PubMed is shown in Table 1.

**Table 1:** Search strategy in PubMed

**Number**	**Search items**
#1	Low back pain [Mesh].
#2	Non-specific low back pain [TIAB].
#3	Non specific low back pain [TIAB].
#4	Lower Back Pain [TIAB].
#5	LBP [TIAB].
#6	NLBP [TIAB].
#7	Acute lumbar sprain[TIAB].
#8	#1 or #2 or #3 or #4 or #5 or #6 or #7
#9	Acupuncture [Mesh].
#10	Acupuncture [TIAB].
#11	Electroacupuncture [Mesh].
#12	Electroacupuncture [TIAB].
#13	Manual acupuncture [TIAB].
#14	Filiform needle [TIAB].
#15	Acupuncture point [TIAB].
#16	Acupoint [TIAB].
#17	Auricular acupuncture[TIAB].
#18	Warm acupuncture[TIAB].
#19	Fire acupuncture [TIAB].
#20	#9 or #10 or #11 or #12 or #13 or #14 or #15 or #16 or #17 or #18 or #19
#21	Meta-analysis [PT].
#22	Meta-analysis [TIAB].
#23	Systematic review [TIAB].
#24	#21 or #22 or #23
#25	#8 and #20 and #24

### Literature Screening

According to the pre-developed standardized search strategy, two researchers (YWX, WX) searched databases and exported the retrieved literature titles into Endnote X8 software. After removing the duplicate data, two researchers independently screened the titles and abstracts of the retrieved literature according to the inclusion and exclusion criteria and deleted unrelated literature. Then, those that matched the requirements literature were downloaded and read to determine whether they met the inclusion criteria. Any disagreements were reviewed and adjudicated by a third reviewer (ZW).

### Data Extraction

In each SR/MA, the following data were extracted: title, author, published year, RCT included in the study, sample size, the intervention of the experimental group, the intervention of the control group, risk of bias (RoB) evaluation tool, outcomes, and principal conclusions. Two researchers (YWX and WX) undertook data extraction, and conflict resolution was achieved by discussion and consultation with a third author (ZW).

### Assessment of Methodological Quality

Two reviewers (YWX and LHZ) independently assessed the methodological quality of SRs/MAs using the AMSTAR 2 comprising 16 items. Seven (items 2, 4, 7, 9, 11, 13, and 15) are critical, while the remainder are non-critical. Each item is evaluated as “Yes,” “Partially Yes,” or “No.” Based on the evaluation results and the criticality of the entries, it categorized the overall reliability of the evaluation into four categories: high, moderate, low, and critically low. The quality of the methodology was rated as “high” with no or one non-critical weakness, “moderate” with multiple weaknesses and no critical flaws, “low” with one critical flaw and unlimited non-critical weaknesses, or “very low” with more than one critical flaw [12, 13]. 

### Assessment of Report Quality

By the PRISMA statement, the quality of the reports of the SRs/MAs that were included was assessed [14]. Two reviewers (YWX and WX) independently evaluated the report quality of SRs/MAs using the PRISMA statement comprising 27 items. The PRISMA statement list consists of 27 items, covering 7 aspects of SRs/MAs, including title, abstract, introduction, methods, results, discussion, and other information. Each item is described with “yes,” “partially yes,” and “no,” representing a complete report, a partially compliant report, and a no report. The completion of each item is presented as a ratio. Discrepancy items following the evaluation were discussed and finally agreed upon by a third evaluator (ZW).

### Assessment of Quality of Evidence Bodies

The quality of primary outcomes of included SRs/MAs was evaluated by the GRADE system [15]. Two authors (YWX and LLP) utilized the GRADE system to assess the quality of evidence bodies of the outcome measures included in the SRs/MAs based on five factors: limitations, inconsistencies, indirectness, inaccuracy, and publication bias. Evidence quality was categorized as high, moderate, low, and very low. Detailed information on the ratings can be found in papers published by the GRADE working group [8]. Cross-checking was done after the analysis was completed, and disputes were adjudicated by a third party (ZW).

## Results

### Search Results

A total of 927 potential studies were identified through initial database searching. Five hundred thirty-one studies were excluded for duplication, and 343 were excluded based on their title or abstract. Seventeen of the remaining 40 studies were excluded after examining the full text [16–32]. Characteristics of articles excluded after full reading are shown in Appendix. After being reviewed by two reviewers independently, 23 SRs/MAs on acupuncture for LBP were included [5, 33–54]. 

**Fig. 1:**
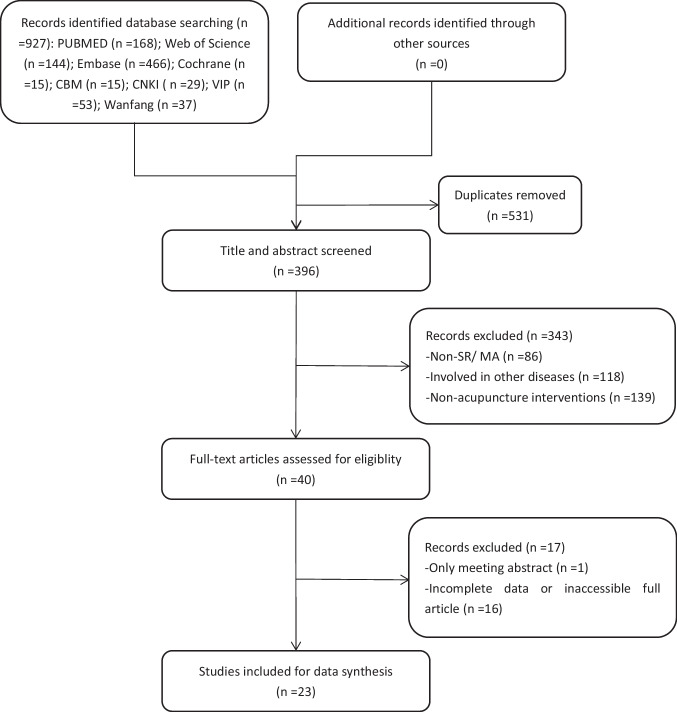
Flow diagram of literature searching

A flow chart of the study selection process is presented in Fig. 1.

### Characteristics of the Included Literatures

Twenty-three SRs/MAs met the inclusion criteria, with a concentration of publication years from 2005 to 2022. Of the 23 SRs/MAs, 11 (47.83%) were published in Chinese, and 12 were published in English. In 13 studies, interventions were only acupuncture or electroacupuncture. The interventions of 12 studies were acupuncture combined with other treatments. In 16 studies, interventions for the control group included western medicine. The interventions of 14 studies included sham acupuncture, and 6 studies included different acupuncture treatments. All the SRs/MAs were mainly evaluated for total effective rate, pain intensity, and dysfunction. All studies used the Cochrane collaboration’s RoB tool. Seventeen studies mentioned the source of funds, and 6 articles did not mention the funds-related information. The details of the included SRs/MAs are summarized in Table 2.

### Assessment of Quality of SRs/MAs

***Assessment Methodological Quality of SRs/MAs by AMSTAR 2***

Using the methodological quality evaluation of the AMSTAR 2 scale, it was shown that the methodological quality of one SR (1/23, 4.35%) was medium, one MA (1/23, 4.35%) was of low quality, and the other SRs/MAs (21/13, 91.30%) were “extremely low.” The research questions and inclusion criteria for all 23 SRs/MAs included the components of PICO. All used an excellent technique for assessing the risk of bias (RoB) in individual studies that were included and used appropriate methods for the statistical combination of results. Most SRs/MAs did not provide a protocol in the explicit statement (21/23, 91.30%), did not explain why RCTs were chosen (20/23, 86.96%), did not report on the sources of funding for the studies included in the SRs/MAs (22/23, 95.65%), and did not provide a list of excluded studies and justify the exclusions (21/23, 91.30%). Only two SRs/MAs provided a comprehensive literature search strategy (2/23, 6.70%), three SRs/MAs described the included studies in adequate detail (3/23, 13.04%), and two SRs/MAs did not use study selection and data extraction in duplicate (2/23, 6.70%). Two SRs/MAs did not assess the potential impact of RoB in individual studies (2/23, 6.70%). Six SRs/MAs explained or discussed whether the risk of bias of the included studies was considered for each study outcome (6/23, 26.08%), 14 SRs/MAs provided a satisfactory explanation for any heterogeneity observed in the results (14/23, 60.87%), 15 SRs/MAs assessed the likelihood of publication bias (15/23, 65.22%), and 17 SRs/MAs reported potential sources of conflict of interest (17/23, 73.91%). The results of the methodological quality of included SRs/MAs are presented in Table 3. Table 4 summarizes the methodological quality of the included SRs/MAs by 16 AMSTAR 2 items.

#### Assessment of Quality of Report by PRISMA Statement

**Fig. 2:**
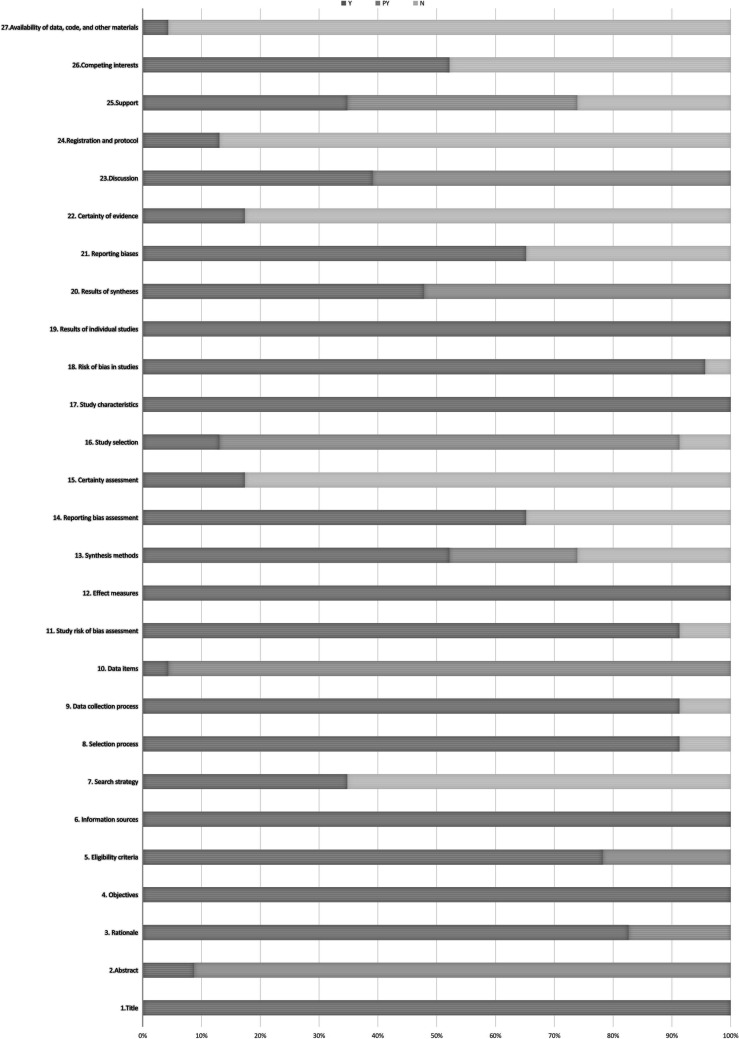
A summary of the PRISMA findings. Y, yes (a complete report); PY, partially yes (a partially compliant report); N, no (no report)

Reporting quality was assessed using the PRISMA statement for 23 SRs and MAs. No study reported all the items of the PRISMA. All 23 SRs/MAs were fully reported with titles, objectives, information sources, effect measures, study characteristics, and results of individual studies. The abstract section had only two SRs/MAs who submitted complete abstracts (2/23, 8.69%). In the introduction, 19 SRs/MAs described the rationale for the SR/MA in the context of existing knowledge and how it complements existing theory (19/23, 82.61%). In the section on the methods, 18 SRs/MAs specified the inclusion and exclusion criteria for the review and how studies were grouped for the syntheses (18/23, 78.26%); 8 SRs/MAs reported fully on the search strategy, including any filters and limits used (8/23, 34.78%); 21 SRs/MAs elaborated on the selection process and data collection process (21/23, 91.31%); only one SR summarized the data items altogether ultimately (1/23, 4.35%); 21 SRs/MAs specified the methods used to assess the risk of bias in the included studies and pertinent details (21/23, 91.31%); 12 SRs/MAs provided complete synthesis methods (12/23, 52.17%); 15 SRs/MAs reported bias assessment (15/23, 65.22%); and 4 SRs/MAs described a method used to assess certainty (or confidence) in the body of evidence for an outcome (4/23, 17.39%). In the part of the results, only 3 SRs/MAs reported fully on the study selection (3/23, 13.04%), 22 SRs/MAs presented assessments of risk of bias for each included study (22/23, 95.65%), 11 SRs/MAs reported the results of syntheses completely (11/23, 47.83%), 15 SRs/MAs presented assessments of risk of bias due to missing results (arising from reporting biases) for each synthesis assessed (15/23, 65.22%), and only 4 SRs/MAs presented assessments of certainty (or confidence) in the body of evidence for each outcome assessed (4/23, 17.39%). In the discussion section, 9 SRs/MAs offered complete discussion (9/23, 39.13%). In the section on other information, 3 SRs/MAs provided registration and protocol information (3/23, 13.04%), 8 SRs/MAs thoroughly described sources of financial or non-financial support for the review and the role of the funders or sponsors in the review (8/23, 34.78%), and 12 SRs/MAs provided competed for interests’ information (12/23, 52.17%). Only one SR reported the availability of data, code, and other materials (1/23, 4.35%). Table 5 and Fig. 2 provide PRISMA statement for each SR/MA.

#### Assessment of Quality of Evidence Bodies by GRADE

The quality of evidence generated by the included SRs/MAs was evaluated according to the GRADE, involving 255 evidence bodies. Of the 255 bodies of evidence generated, 88 (34.51%) were of low quality, 154 (60.39%) were of very low quality, and 13 (5.10%) were of moderate quality. Publication bias was the most common factor in downgrading evidence quality, followed by limitation, imprecision, inconsistency, and indirectness. The details are given in Table 6.

## Primary Pain Intensity Outcome—VAS

Twenty-one SRs/MAs with a total of 74 bodies of evidence used the VAS to evaluate the efficacy of acupuncture in treating LBP, of which 10 were of moderate quality, 28 were of low quality, and 36 were of very low quality.

## Primary Dysfunction Outcome—RMDQ

A total of 12 SRs/MAs conducted 38 bodies of evidence using the RMDQ to assess the efficacy of acupuncture for the treatment of LBP, of which one evidence body was of moderate quality, 11 were of low quality, and 26 were of very low quality.

## Total Effective Rate

Nine SRs/MAs with a total of 20 bodies of evidence used the total effective rate to evaluate the efficacy of acupuncture in treating LBP, of which 12 were low quality and 8 were very low quality.

## Discussions

### Discussion Summary of Main Findings

The purpose of an SRs/MAs overview is to reevaluate a comprehensive collection of studies relating to the same disease or health problem, give clinical work guidance, and provide the basis for developing clinical guidelines. This overview synthesized evidence on the effectiveness and safety of acupuncture for LBP from 23 SRs/MAs. The main conclusion was that acupuncture is more effective than a placebo, western medicine, sham acupuncture, physical therapy, usual care, or TENS in treating LBP. In addition, acupuncture combined with other treatments is more effective than acupuncture alone at relieving LBP. At the same time, different acupuncture treatments (different acupoints, manipulations, courses of treatment, etc.) have different efficacy in relieving LBP. However, regarding the appraisal results of AMSTAR 2, PRISMA statement, and GRADE, the methodological, report, and evidence quality of most SRs/MAs could have been better. These findings suggest that the results of all existing SRs/MAs may overestimate the actual effects of acupuncture. Hence, further studies with an improved methodological design are needed to accurately determine the actual effectiveness and safety of acupuncture in the management of LBP.

### Methodological Quality of Included SRs/MAs

The AMSTAR 2 methodological quality evaluation results revealed common deficiencies in the included SRs/MAs. Only two SRs/MAs provided a study protocol in the explicit statement, and in particular, none of the Chinese SRs/MAs had a detailed study protocol before implementation. Whereas SR/MA is a form of observational research, it is essential to remain prospective. Developing a study protocol before the start of SR/MA can reduce bias and increase the rigor of SR/MA. Twenty of the SRs/MAs did not state the reasons for specifying the type of included studies, and the selection of research design types in SRs/MAs should not be arbitrary and should follow some strategies or rules. Most SRs/MAs did not perform supplementary searches or complete reports, nor did they search the gray literature. They mainly provided search terms without presenting specific search strategies, all of which may have contributed to publication bias. Like item 2, only two SRs/MAs in item 7 provided a list of excluded studies and justified the exclusions. Most SRs/MAs only briefly discussed the studies’ screening process or only explained the reasons for exclusion, which reduced the credibility and rigor of the screening literature. Twenty-two SRs/MAs did not report funding sources and did not provide conflicts of interest. Since corporate-funded research results are more biased towards funders and less likely to be published, information about funding extracted from included studies can be used to determine the impact on research results. Failure to provide funding sources or conflicts of interest will make it difficult for researchers to assess possible conflicts of interest, resulting in the human impact of evaluation results and the risk of bias.

### Quality of Report of Included SRs/MAs

According to the results of the PRISMA evaluation presented in this overview, the quality of reporting of the SRs/MAs could have been better. Methods, results, and other information were inadequately reported in most of the SRs/MAs included. In the method section, 15 SRs/MAs failed to describe their search strategy completely. In contrast, the latest PRISMA statement requires the presentation of the comprehensive search strategy for all databases, registration platforms, and websites, including the filters and qualifiers used. A complete search strategy facilitates the reader in assessing the comprehensiveness of the search, increases transparency and reproducibility in the production of systematic evaluations, and facilitates its updating. Nineteen SRs/MAs did not describe the methods used to evaluate the quality of the evidence for each outcome. This item was added to PRISMA 2020, which requires the author to describe the methods used to assess the quality of the outcome evidence (or its credibility). In the results section, 19 of the 19 SRs/MAs do not present assessments of the quality of evidence for each outcome. This item echoes item 15 of the method section, all of which require the use of GREAD or other methods for grading the quality of evidence for each outcome. In the other information section, 20 SRs/MAs did not fully report registration and protocol information. The following sub-items comprise this item:

- Provide the registration information.
- Describe how the review protocol can be accessed or state that no protocol has been prepared.
- Indicate and explain any changes to the information provided at registration or in the protocol.

Providing this information is helpful for readers to judge which information is pre-planned and which information is finally reported and to assess whether the bias will cause the risk of bias. Twenty-two SRs/MAs did not provide all the necessary data, codes, code, and other materials. Most SRs/MAs were unaware of the need to provide this information. Sharing data, analyzing code, and other materials may help us to reuse that data, discover data errors, regenerate reports, and understand analytical methods.

**Table 2:**
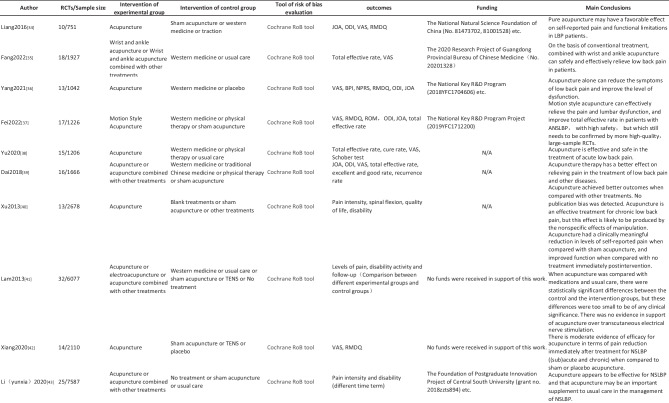
Characteristics of the included SRs/meta-analysis

### Quality of Evidence of included SRs/MAs

From Table 6, a total of 255 outcomes were included in this overview, most of which are subjective evidence, such as total effective rate, recurrence rate, VAS, NRS, MPQ, RMDQ, ODI, HFAQ, SF-12, and SF-36. These outcomes were based on participants’ subjective feelings, with some limitations. The results of the GRADE evaluation suggest that most of the evidence quality included in the outcomes is low or very low, and only a few reach moderate quality, a result that indicates that the credibility of the body of evidence generated by the included SRs/MAs may differ from the clinical reality and should be referred to with caution. The most significant factor contributing to downgrading was publication bias. Publication bias was primarily evident in the asymmetric funnel plots, the inadequately narrow confidence intervals, and the inclusion of studies that did not meet the sample size estimation requirements for clinical trials or studies with potential publication bias. The second was the limitation, which showed that the methodological design of the RCTs (randomization, group concealment, blinding, etc.) was heavily biased, with most RCTs only mentioning randomization but not describing the specific randomization method and failing to conceal allocation. Only a few studies mentioned blinding, and most only utilized single blinding. Imprecision was mainly due to wide confidence intervals, inadequate sample sizes of included studies, and lack of overlap between CIs. Furthermore, the inconsistency of the results can be seen in the slight overlap of confidence intervals between studies, the small *p*-values for heterogeneity tests, and the significant heterogeneity (I^2^ > 50%).

**Table 3:**
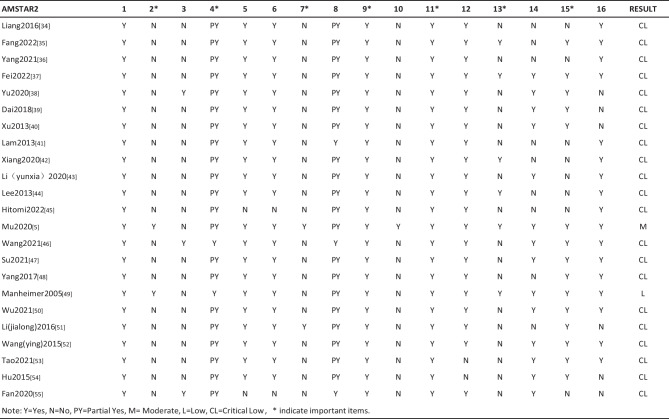
Methodological quality of the included SRs/meta-analysis by AMSTAR 2

### Limitations

There are certain limitations in this reevaluation:

1. Only SRs/MAs published in Chinese and English were included. No other minor languages and no unpublished literature were retrieved, which to some extent, limits the exposure of negative results and leads to publication bias.
2. There was an overlap in the RCTs of the included SRs/MAs, which may lead to double counting results. Due to the generally low quality of evidence included in the SRs/MAs, the results are very biased.
3. Only a few SRs/MAs evaluated the effectiveness and safety of LBP by acupuncture methods. The acupuncture methods mentioned in the remaining SRs/MAs did not provide sufficient evidence due to unclear comparisons or limited sample size.
4. AMSTAR 2 and GRADE scale assessments by different researchers might be biased, even if their assessments were cross-checked and examined further by different researchers.

**Table 4:**
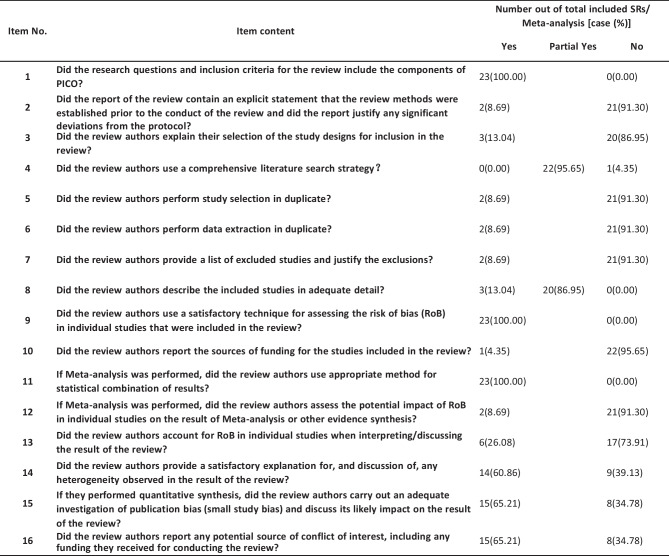
Summary of SR/meta-analysis methodological quality by AMSTAR 2 items

## Conclusion

In summary, the available evidence shows that acupuncture has certain advantages in treating LBP. However, the overall methodological quality of SRs/MAs and the quality of evidence for outcomes still need to be improved. Accordingly, it is recommended that researchers design the study protocol scientifically and rationally from the start and strictly follow a multicenter, large sample, randomized, double-blind experimental design to reduce bias from the source of evidence. At the same time, system evaluators are trained in methodological and quality assessment and other evidence-based competencies and strictly follow the corresponding quality assessment criteria when implementing SRs/MAs. Improving the quality of SRs/MAs will provide more evidence-based medical evidence for users of the evidence for definitive conclusions.

**Table 5:**
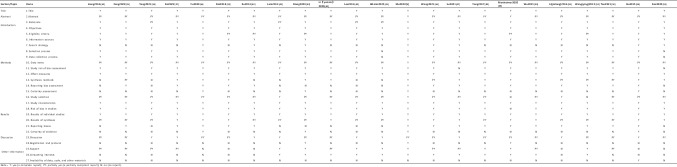
Assessment of quality of report by PRISMA statement

*Y* yes (a complete report), *PY* partially yes (a partially compliant report), *N* no (no report)

**Table 6:**
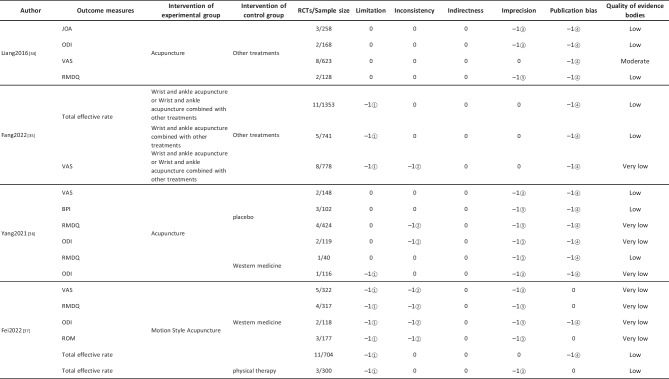

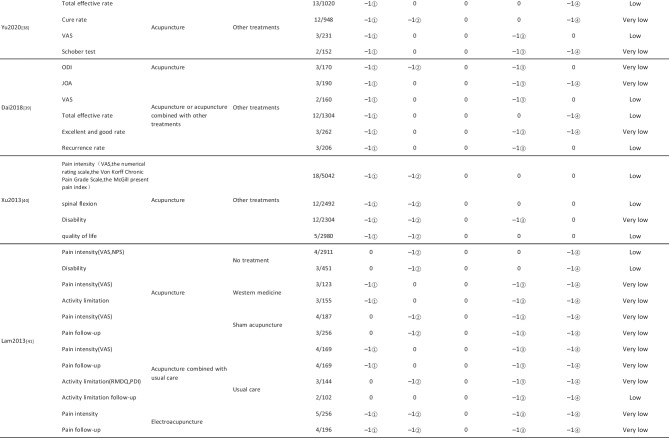

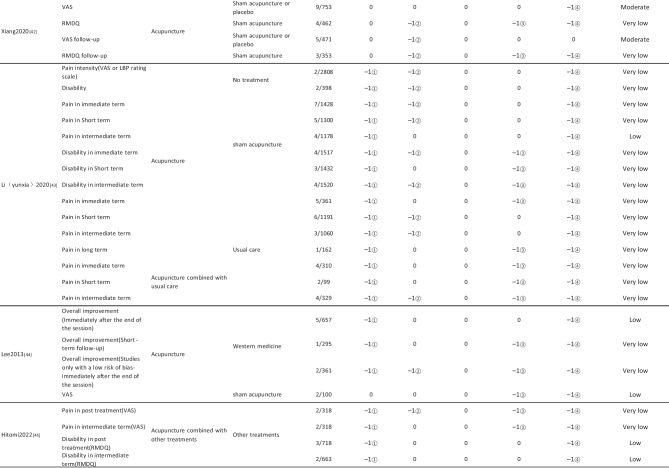

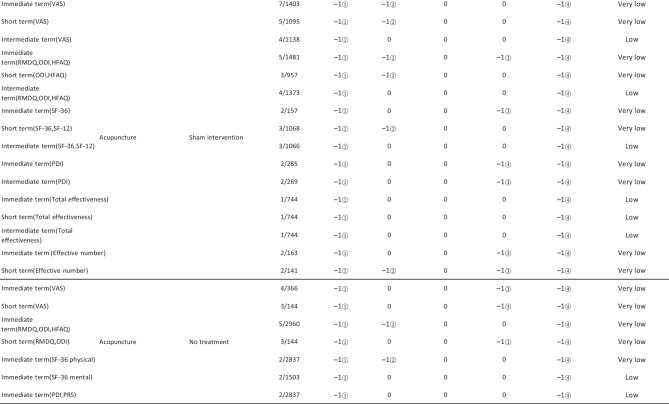

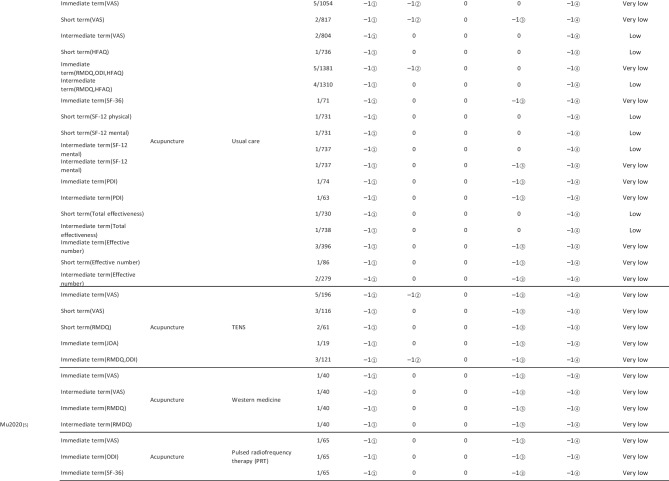

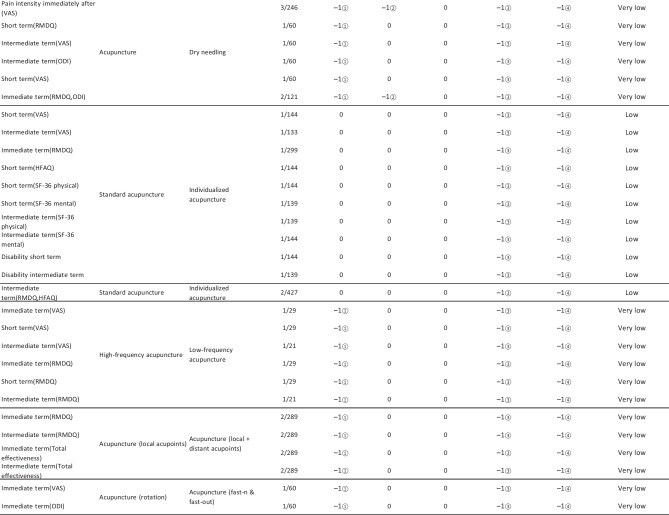

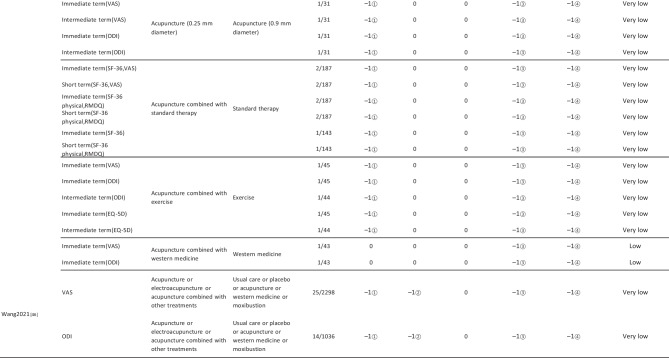

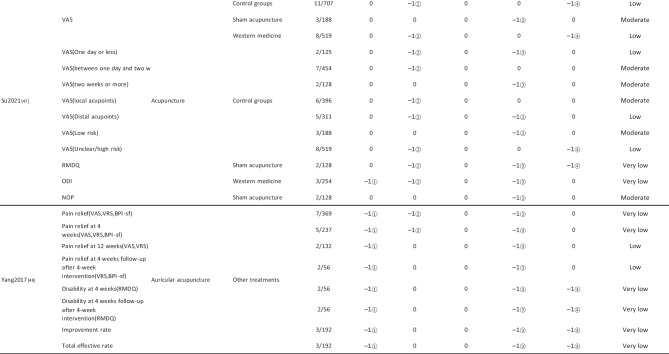

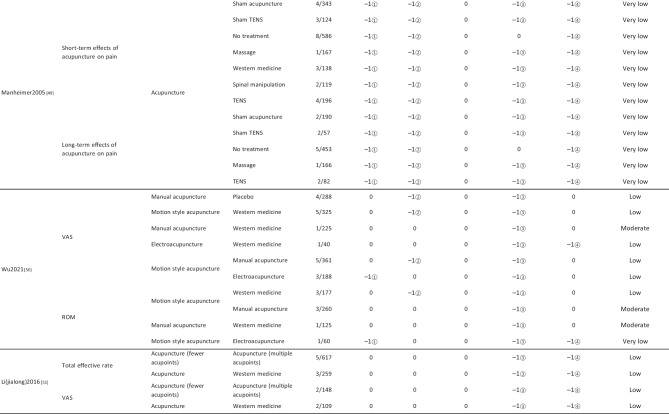

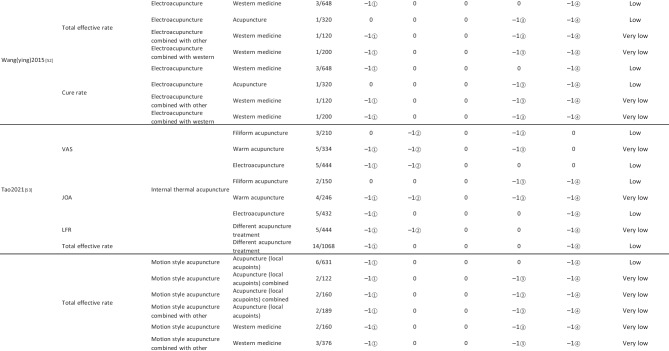

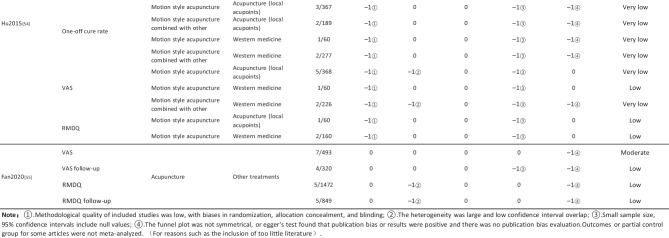
Assessment of quality of evidence bodies from included SRs/meta-analysis by GRADE in outcome level

## Data Availability

You can contact the corresponding author for the data.

## Appendix: Characteristics of articles excluded after full reading

**Table:**

**Article**	**Exclusion reasons**
Baroncini et al. [16]	Incomplete data
Zeng et al. [17]	Incomplete data
Liu [18]	Incomplete data
Wang et al. [19]	Incomplete data
Johnston et al. [20]	Incomplete data
Huang et al. [21]	Incomplete data
Trigkilidas [22]	Incomplete data
Tulder et al. [23]	Incomplete data
Wen et al. [24]	Incomplete data
Yuan et al. [25]	Incomplete data
Fuentes et al. [26]	Inaccessible full article
Sung et al. [27]	Incomplete data
Henderson [28]	Incomplete data
McIntosh and Hall [29]	Inaccessible full article
Liang et al. [30]	Incomplete data
Kong et al. [31]	Incomplete data
Yang et al. [32]	Only meeting abstract

## Abbreviations

LBP: Low back pain
SRs: Systematic reviews
MAs: Meta-analyses
CBM: Chinese Biomedical Literature Database
CNKI: China National Knowledge Infrastructure
NLBP: Non-specific low back pain
AMSTAR 2: A Measurement Tool to Assess systematic Reviews, revised edition
PRISMA: The Preferred Reporting Items for Systematic reviews and Meta-Analyses
GRADE: The Grading of Recommendations Assessment, Development and Evaluation
RCTs: Randomized control trials
TENS: Tanscutaneous electrical nerve stimulation
VAS: Visual Analogue Scale
JOA: Japanese Orthopaedic Association Scores
NRS: Numerical Rating Scale
NPRS: Numerical Pain Rating Scale
RMDQ: Roland-Morris Disability Questionnaire
ODI: Oswestry Disability Index
PDI: Pain Disability Index
HFAQ: Hanover Functional Ability Questionnaire
BPI: Brief Pain Inventory
CPGS: Von Korff Chronic Pain Grade Scale
MPQ: McGill Pain Questionnaire
BPI-SF: Brief Pain Inventory Short Form
NOP: Numbers of pills
ROM: Range of motion
LFR: Lumbar flexion range
SF-12: The short-form-12 health survey
SF-36: The short-form-36 health survey
PRS: Pain Rating Scale
EQ-5D: EuroQol 5D
RoB: Risk of bias
